# Multi-Scenario Landscape Ecological Risk Simulation for Sustainable Development Goals: A Case Study on the Central Mountainous Area of Hainan Island

**DOI:** 10.3390/ijerph19074030

**Published:** 2022-03-29

**Authors:** Nianlong Han, Miao Yu, Peihong Jia

**Affiliations:** School of Public Administration, Hainan University, Haikou 570228, China; nlhan@hainanu.edu.cn (N.H.); yumiao@hainanu.edu.cn (M.Y.)

**Keywords:** SDGs, landscape ecological risk, PLUS model, central mountainous area (CMA), Hainan Island

## Abstract

The sustainable development goals (SDGs) of the United Nations are focused on regional development and ecological security. Based on these SDGs, quantitative regional landscape ecological risk assessment is significant to realize regional sustainable development. This study took the central mountainous area (CMA) of Hainan Island as the research area, and combined SDGs and a patch-generating land-use simulation (PLUS) model to analyze multi-scenario land-use change and landscape ecological risk simulation. The study results show that the low ecological risk areas are located in the central hinterland of the CMA, and the high ecological risk areas are located on the northern and southern edges, with strong disturbances from human activities. The construction land in the CMA expanded drastically from 2010 to 2018, mainly invading forestland and grassland, leading to landscape fragmentation, which was the main cause of the increased ecological risk in the CMA landscape. The future multi-scenario simulations for SDGs show that under the scenario of natural development and economic development, the construction land and water area will significantly expand and the forest land will be dramatically reduced. Under the ecological protection scenario, the expansion of construction land will be restrained, and the area of forest land will increase. The results showed that the landscape ecological risks in the three simulated scenarios would be higher than in 2018, but the increase in the landscape ecological risks under the ecological protection scenario would be relatively slight. Forest land plays an essential role in maintaining the ecological security of the CMA. The expanding construction land in the CMA has led to landscape fragmentation and increased ecological risk. Therefore, it is necessary to protect the forest land in the CMA. In addition, construction and development should be limited in high-risk areas. Although the adoption of the ecological conservation scenario favors regional sustainability, it is still necessary to improve ecological protection policies such as ecological compensation to ensure the realization of other SDGs.

## 1. Introduction

The rapid development of global urbanization has aggravated the deterioration of the environment, and sustainable development has attracted widespread attention worldwide [1]. In 2015, the United Nations proposed global sustainable development goals (SDGs), which mainly included 17 universal and comprehensive primary goals of sustainable development (Table 1), aiming to achieve the goals of sustainable development in three dimensions: economic development, promoting a beautiful environment, and social progress [2]. However, there are differences in development and various practice paths in different countries [3]. Therefore, it is essential to research regional development combined with SDGs and take proper measures to promote the accomplishment of SDGs in China.

The landscape’s ecological risk can reflect environmental issues caused by the interaction between nature and human beings. It can also effectively guide the management and optimization of regional land use. Landscape ecological risk assessments pay more attention to spatial-temporal heterogeneity and scale effects than traditional ecological risk assessments [4]. This type of risk assessment reflects the negative impacts of human activities or natural environmental changes on the ecosystem from a spatial perspective [4,5]. The landscape is the optimal scale to understand the dynamic relationship between society and nature, achieve the sustainability of ecosystem services, and promote human well-being [6]. Furthermore, benchmarking landscape ecological risks with SDGs indicators shows consistency and leadership in ecosystem protection [7]. Thus, combining landscape patterns of land-use types and SDGs can promote the understanding and improvement of the dynamic relationship between ecosystem services and human well-being.

The central mountainous area (CMA) of Hainan Island, China, is rich in biodiversity and forest resources. It is the source area of Hainan Island’s major rivers and the core water conservation area on Hainan Island, so it plays an essential role in maintaining the island’s ecological balance and sustainable development [8]. The changes in its ecological system structure and service functions are susceptible to local social and economic development [9]. In recent decades, rapid economic development and frequent human activities in Hainan have caused drastic changes in landscape patterns. Human activities have a dual impact on ecological risks: on the one hand, activities such as urban expansion, farmland cultivation, and orchard planting will damage the ecology. On the other hand, vegetation restoration projects (such as returning farmland to forest, and grassland and ecological forest construction) have a certain positive effect on increasing vegetation coverage and reducing ecological risks. Therefore, revealing and evaluating the impact of human disturbance on ecological risk is beneficial to regional ecological sustainability management. Moreover, it is of great scientific significance to simulate the response of landscape ecological risk to landscape pattern changes in combination with SDGs.

Currently, the simulation models of land-use change mainly include future land use simulation (FLUS), conversion of land use and its effects (CLUE-S), and cellular automata and Markov chain (CA-Markov) models. CLUES and CA-Markov models often train and estimate the conversion probability of each landscape type individually, ignoring the connection between landscape types [10,11,12]. Although the FLUS model solves this problem, it cannot reveal how driving factors lead to land-use change [13]. The patch-generating land-use simulation model (PLUS) overcomes the above shortcomings: it simulates land-use change from the perspective of patches to achieve more reliable landscape pattern metrics with respect to the real-world landscape, which aids the accuracy of ecological risk assessments based on landscape pattern [14]. Therefore, the purpose of this research was to: understand the spatio-temporal response relationship between landscape ecological risk and land-use change of the CMA; explore the determining factors of CMA land-use change; consider this in relation to SDGs and land-use prediction models to simulate the CMA land-use and landscape ecological risk changes under different scenarios; and finally to seek a sustainable development method in the CMA of Hainan Island, providing a solid case study for the regional optimization of SDGs.

## 2. Study Area and Materials

### 2.1. Study Area

**Figure 1:**
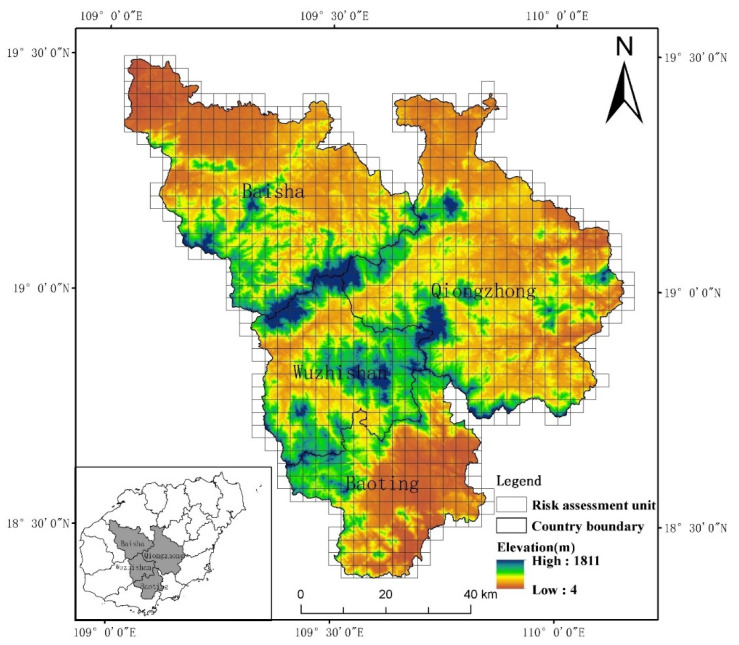
The location of the central mountainous area and risk assessment unit division.

The CMA is located in the central and southern part of Hainan Island (Figure 1), with a total area of about 7115.41 km^2^. Its administrative districts include Wuzhishan City, Qiongzhong, Baisha, and Baoting County. The main land cover types include grassland, low mountain hills, and tropical rain forests. In 2010, the CMA of Hainan Island was identified as the national key ecological function area. In the same year, the International Tourism Island policy was implemented, resulting in rapid economic and social development and significant urban expansion [15]. Because of the excessive use of resources and the lack of effective management, the fragile ecological environment in the central mountainous area has been damaged. Since 1999, a large area of economic forests and crops such as pulp forests, rubber forests, and areca forests have been planted in the CMA, resulting in a large reduction in natural forests. From 2001 to 2010, about 13.02% of the natural tropical forest was converted to pulp and paper plantations in the CMA [16]. The CMA has rich water resources and outstanding water quality, which is suitable for aquaculture. However, due to the limited conditions of terrain, aquaculture operations are scattered [17]. Therefore, the impact of human activities has caused great pressure on landscape ecological risk in the CMA. Currently, with the promotion of the Hainan free-trade port and the goal of the construction of an ecological civilization, the coordinated and sustainable development of the social economy and ecological security in the CMA is urgent.

### 2.2. Materials

The research data were divided into landscape risk assessment and prediction model driving factor data. Landscape ecological risk assessment data mainly included land-use data from 2000, 2010, and 2018 at a resolution of 30 m and were obtained from the Resource and Environmental Science Center of the Chinese Academy of Sciences (www.resdc.cn, 1 December 2020). The driving factor data of the PLUS model included natural factors and socio-economic data. Natural factors including elevation, slope, and road data were obtained from the National Basic Geographic Information Center (www.ngcc.cn/ngcc, 1 December 2020). The dataset of distance to rivers and town centers were processed and extracted according to the land-use data. Socioeconomic data including population and GDP were obtained from the Hainan Statistical Yearbook.

## 3. Methodologies 

In this study, the grid analysis method was used to calculate the landscape ecological risk index of each grid in different periods using Fragstats 4.2 software (http://www.umass.edu/landeco/research/fragstats, Amherst, MA, USA), and kriging interpolation was used to obtain the spatial and temporal distribution of landscape ecological risk in the CMA. Finally, the PLUS model was used to simulate the change in future land use and landscape ecological risk.

### 3.1. Landscape Ecological Risk Assessment

Combined with the actual situation of the CMA and according to the principle of 2–5 times the average area of landscape patches in the study area [18], the study area was divided into 907 evaluation units with 3 km × 3 km grid by sampling at equal intervals (Figure 1). Through the proportion of land-use types and their landscape loss index, the landscape ecological risk index (ERI) was constructed to evaluate the ecological risk of each unit [19]. The formula is as follows:

(1) $$ERI_{k}=\overset{N}{\underset{i=1}{\sum }}\frac{A_{ki}}{A_{k}}\times R_{i},\;$$

where *N* is the number of land-use types, i is the land-use type, *A_ki_* is the area of land-use type *i* in the *k*-th evaluation unit, *A_k_* is the area of the *k*-th evaluation unit, and *R_i_* is the loss degree index of a certain landscape type i, which reflects the degree of the loss of natural attributes of ecosystems represented by different landscape types when they are disturbed by natural and anthropogenic causes [20,21]. The formula for *R*_i_ is:

(2) $$R_{i}=E_{i}\times V_{i},$$

where *E_i_* is the landscape disturbance index, which reflects the loss degree of different landscape types after being disturbed. The greater the disturbance index, the higher the ecological risk [22]. The formula used to calculate *E_i_* is:

(3) $$E_{i}=aC_{i}+bN_{i}+cF_{i},$$

where *C_i_* is the landscape fragmentation degree; *N_i_* is the landscape separation degree; *F_i_* is the landscape fractal dimension; a, b, and c represent the weights of the above landscape indexes, respectively, where *a* + *b* + *c* = 1. The values of 0.5, 0.3, and 0.2 were assigned to *a*, *b*, and *c*, respectively, according to relevant studies [23]. The relevant calculations and interpretations are shown in Table 2. 

### 3.2. PLUS Model

The PLUS model integrates a rule-mining framework based on a land expansion analysis strategy (LEAS) with a cellular automata (CA) model based on multi-type random seeds (CARs), which can better reveal the effects of various land-use changes [14].

The LEAS extracted the land expansion between the two periods of land-use data, then randomly extracted samples from the data and employed a random forest classification (RFC) algorithm to explore the relationship between the growth of each land-use type and the driving factors. Finally, the growth probability of each land-use type was calculated.

The CARS combines CA models with patch evolution simulations for multiple land-use types. In the PLUS model, a multi-type random patch-seeding mechanism based on threshold descent was used, which was realized through the calculation process of the overall probability. The mechanism uses the Monte Carlo method to generate the “seed” of change based on the growth probability of each land-use type when the neighborhood effect of a certain land-use type is equal to 0:

(4) $$OP_{i,k}^{d=1,t}=\{\begin{array}{c} P_{i,k}^{d=1}\times \left(r\times \mu_{k}\right)\times D_{k}^{t}\;\;\;\;\;if\;\Omega_{i,k}^{t}=0\;and\;r<\;P_{i,k}^{1}\\ P_{i,k}^{d=1}\times \Omega_{i,k}^{t}\times D_{k}^{t}\;\;\;\;\;\;\;\;\;\;\;\;\;\;\;\;\;\;\;\;\;\;\;\;\;\;\;\;\;\;\;\;\;\;\;\;\;\;all\;others \end{array},\;$$

where $OP_{i,k}^{d=1,t}$ is the overall probability, and$\;P_{i,k}^{d=1}$ is the development probability of land-use type *k* at cell i; $\Omega_{i,k}^{t}$ represents the neighborhood effects of cell *i*, which are the cover ratio of land-use type *k* in the neighborhood; $D_{k}^{t}\;$ is a self-adaptive inertia coefficient that is determined by the current amount of land at iteration t and the target demand of land-use type k; *r* is a random value ranging from 0 to 1; and $\mu_{k}$ is the threshold for the generation of new land-use patches.

The seeds can produce new land-use types and grow into new patches formed by a group of cells that share the same land-use type. In order to control the generation of multiple land-use patches, a diminishing threshold rule for the competitive process is proposed to limit the organic and spontaneous growth of all land-use types. If a new land-use type wins in a round of competition, the decreasing threshold τ is used to evaluate the candidate land-use type c selected by the roulette wheel, as follows:

(5) $$If\;\sum_{k=1}^{N}\left|G_{c}^{t-1}\right|-\sum_{k=1}^{N}\left|G_{c}^{t}\right|<Step\;Then,d=d+1,\;$$

(6) $$\{\begin{array}{c} Change\;\;\;\;\;\;\;\;\;\;\;\;\;\;\;\;\;\;\;\;\;P_{i,c}^{d=1}>\tau \;and\;\;TM_{k,c}\;=1\\ No\;change\;\;\;\;\;\;\;\;\;\;\;\;\;\;\;P_{i,c}^{d=1}\leq \;\tau \;or\;\;\;\;\;TM_{k,c}=0 \end{array}\;\;\;\;\;\tau =\delta^{d\;}\times r1$$

where “Step” is the step size required to simulate land use; *δ* is the decay factor, with a range of 0–1; *r1* is a random value distribution with a mean of 1; and *d* is the number of attenuation steps. Moreover, $TM_{k,c}$ is the transition matrix that defines whether the conversion from land-use type *k* to land-use type *c* is allowed. By using this mechanism, the new land-use patches are allowed to grow under the constraints of the threshold descent rules and the cells with overall probability are most likely to change finally.

## 4. Results

### 4.1. Land-Use Change from 2000 to 2018

**Figure 2:**
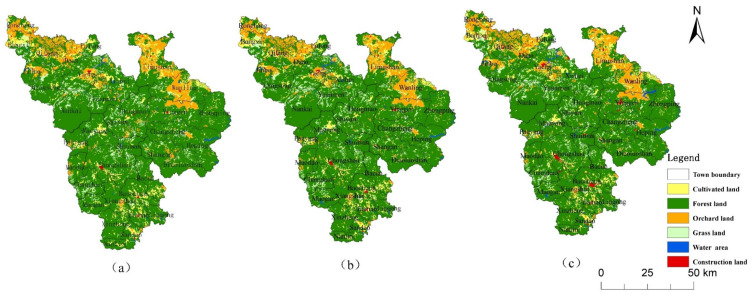
Land-use change in the CMA from 2000 to 2018. (**a**) 2000, (**b**) 2010, (**c**) 2018.

The land-use changes in the CMA from 2000 to 2018 (Figure 2) showed that the area of construction land and the water area increased, the area of cultivated, orchard and grassland decreased, and the forest land increased first and then decreased (Table 3). The area of construction land increased the most, with 32.85 km^2^, followed by water, with an increase of 20.27 km^2^. The increase in water area was mainly due to the increase in the number of reservoirs and ponds, which was related to the increase in aquaculture. Grassland decreased the most, with a decrease of 33.33 km^2^. The main reason for this pattern is that the expansion of towns in the CMA and the promotion of economic forest planting have resulted in large areas of natural forest land, grassland, orchard and cultivated land being replaced by economic forest [26].

**Figure 3:**
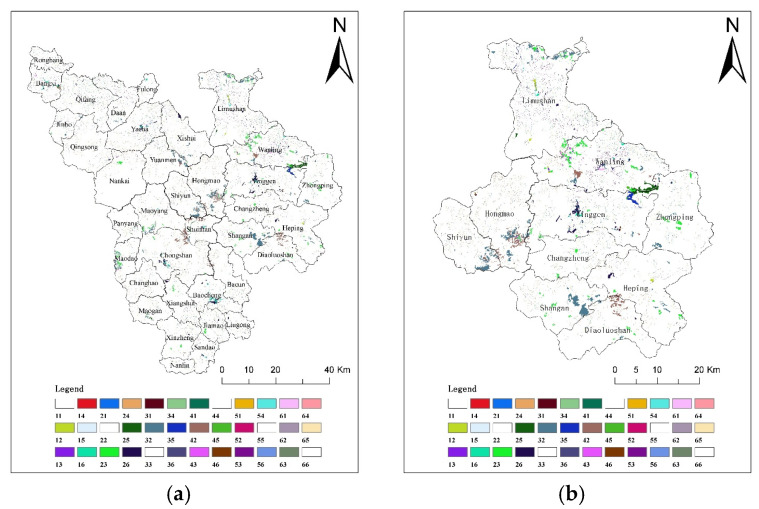
Land-use change from 2000 to 2018. (**a**) CMA (**b**) Qiongzhong (1—cultivated land; 2—forest land; 3—orchard; 4—grassland; 5—water area; 6—construction land).

It can be seen from the land-use change that Qiongzhong County, located in the CMA, has a high intensity of human activities, and the land-use change there was larger than in other places (Figure 3a,b). Therefore, we evaluated the landscape ecological risk in Qiongzhong County and compared it with the CMA.

### 4.2. Spatiotemporal Variation of Landscape Ecological Risk

The results of the landscape pattern index (Table 4) showed that the fragmentation and separation of forestland and grassland increased in different ranges from 2000 to 2018, showing that during the study period, larger patches of forest land and grassland were segmented, increasing the fragmentation of the landscape. The shape of the landscape became more complex, and the ecological stability was reduced.

The fragmentation and separation of construction land and water areas exhibited a downward trend, which mainly showed that the expansion of towns and water areas connected surrounding patches and made landscape patches more concentrated.

Although the areas of cultivated and orchard land have decreased, their sub-dimensions are increasing, showing that the cultivated and orchard land structure of mountainous terrain is complex and vulnerable to human activities, and has low ecological stability. The loss of construction land decreased the most, showing that urban construction has expanded in an orderly manner [27]. The loss of other types such as grassland, forestland, and cultivated land has increased, showing that these types are susceptible to interference from human activities. Among them, the loss of grassland increased the most, which showed that the effects of interference due to human activities on grassland have increased. In general, forest land accounted for about 74% of the entire CMA, but its patch ratio was only about 10%, showing that its patch integrity was high, and the fragmentation was low. Therefore, forest land plays an important role in maintaining the ecological risk of the CMA.

**Figure 4:**
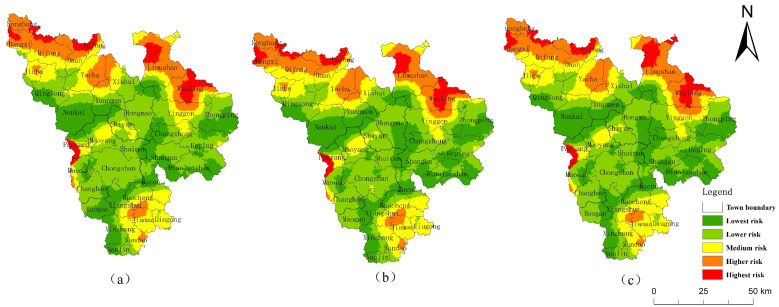
Spatial distribution of landscape ecological risk in the CMA. (**a**) 2000, (**b**) 2010, (**c**) 2018.

In 2000, 2010, and 2018, the ERI values of the CMA were 0.09347, 0.09318, and 0.09364, respectively. The ecological risk decreased first and then increased. The ERI of the central mountainous area was classified into five grades using the natural breakpoint method (Figure 4): highest ecological risk area (ERI > 0.1315); higher ecological risk area (0.1093 < ERI ≤ 0.1315); medium ecological risk area (0.0906 < ERI ≤ 0.1093); lower ecological risk area (0.0761 < ERI ≤ 0.0906); and lowest ecological risk area (ERI ≤ 0.0761). The results showed that the ecological background of the CMA is good, and the region is dominated by mainly lower and lowest ecological risk areas. The high ecological risk areas were mainly distributed in the west and northeast of Baisha County (Rongbang Town, Banxi Town, Qifang Town, Yacha Town, Fulong Town, Yacha Town, and Xishui Town), the north of Qiongzhong County (Limu Mountain Wanling Town, and Yinggen town), the southeast of Baoting County (Liugong Town, Sandao Town, Jiamao Town), and the west of Wuzhishan County (Panyang Town).

From 2000 to 2010, the urban expansion in the CMA was not significant, and the implementation of the policy of returning farmland to forest resulted in a reduction in orchard and cultivated land and an increase in forest land, resulting in a slight reduction in the overall ecological risk of the region. From 2010 to 2018, under the influence of the Hainan International Tourism Island policy, the urban expansion in the CMA region was significant, and the urban area increased by 10 times that of 2000–2010. Despite the policy of returning farmland to forests, urban development has still encroached on forest land, resulting in its reduction and increased fragmentation, thus increasing the overall ecological risk in the CMA. Among them, the area of high ecological risk areas increased by 6.25%, mainly in the north of Qiongzhong County.

**Figure 5:**
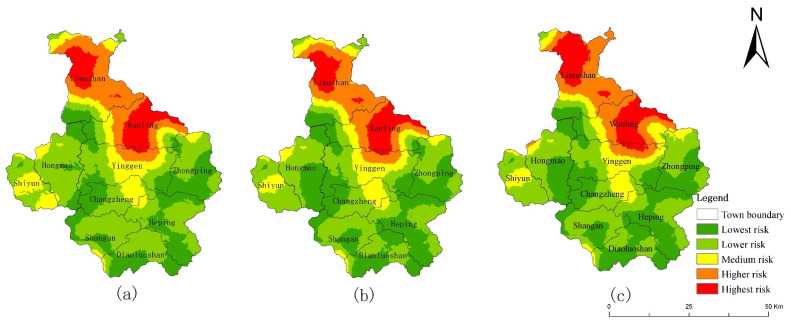
Spatial distribution of landscape ecological risk in Qiongzhong County. (**a**) 2000, (**b**) 2010, (**c**) 2018.

Figure 5 shows the changes in ecological risk in Qiongzhong County from 2000 to 2018. It can be seen that the high ecological risk areas in Qiongzhong County are more obvious. From 2000 to 2018, the overall landscape ecological risk in Qiongzhong County increased from 0.0918 to 0.0924, and the ecological risk in some areas increased obviously.

It can be seen that the ecological risk reduced when the orchard was converted into forest (legend 32 in Figure 3b). Ecological risk increased when the forest was transformed into orchard (legend 23 in Figure 3b), or when the forest was transformed into water (legend 25 in Figure 3b). Especially in the Wanling area, the main change was the conversion of forest to water (Figure 3b), resulting in the ecological risk from low to medium (Figure 5). The changing links between these land-use and ecological risks have a strong indicative effect on the sustainable development of the region. From the analysis of Qiongzhong County, it can be seen that ecological risk changes caused by land-use change have always existed in the CMA, especially in the marginal areas of the CMA (such as Qiongzhong and Baisha counties). If human activities are not restricted, the ecological risk to the landscape may spread further from the edge to the center of the CMA. Therefore, it is necessary to carry out an ecological risk simulation in the CMA in combination with the SDG scenarios to achieve the sustainable development of the region.

### 4.3. Simulation and Analysis of Future Landscape Ecological Risks

#### 4.3.1. Simulation Verification

This study used the PLUS model to predict future land use in 2026 at an interval of 8 years. First, it was necessary to use the land-use changes between 2002 and 2010 to simulate the land use of 2018 and verify the simulation accuracy based on the actual land use. Because of the lack of land-use data in 2002, the recent data from 2000 was used in this study as a substitute. Second, using the LEAS module, the two-period land-use change data was non-uniformly sampled at a sampling rate of 1% and combined with seven driving factors (elevation, slope, road data, distance to the river, distance to the town center, population, and GDP) using the random forest algorithm to calculate the development probability of land-use types. The CA model CARS combined the development of probability, land demand data, transition matrix, and neighborhood weight simulation to obtain land-use changes in 2018. The neighborhood weight value was set according to the expansion ability from strong to weak: construction land was 1, forest land was 0.8, cultivated land and grassland were both 0.5, and water area was 0.1 [28]. The accuracy of the simulation results was verified based on the 2018 land-use data; the kappa coefficient was 0.83, and the overall accuracy was 0.96. The simulation accuracy met the experimental requirements, showing that the PLUS model could be used for the next step of land-use change simulation research.

#### 4.3.2. SDG-Oriented Multi-Scenario Settings

Combined with SDGs, three scenarios of natural development, economic development, and ecological protection were set up to simulate the landscape ecological risks of the CMA in the future. The specific settings of each scenario were as follows.

Under the natural development scenario (NDS), the development goals of SDGs were not considered, no constraint conditions were set, and it was assumed that future land-use change would continue the development trend of 2000–2018.

The economic development scenario (EDS) emphasizes that the goal is to prioritize the development of the economy, combined with the goal of “building disaster-resistant infrastructure, promoting inclusive and sustainable industrialization and promoting innovation” proposed by SDG 9 and “building inclusive, safe, disaster-resistant and sustainable cities and human settlements” proposed by SDG 12 [2]. In the future, land-use changes will result in an increased demand for construction land, so the probability of converting cultivated, orchard, forest, grassland, and water into construction land will be increased by 40%.

The purpose of the ecological protection scenario (EPS) is to strictly protect ecological land, and the CMA is an important ecological functional area of the country. Protecting forest resources, especially tropical rainforests, is of great significance. According to SDG 15, with the goal of “protecting, restoring and promoting sustainable development, using terrestrial ecosystems, sustainable forest management, combating desertification, stopping and reversing land degradation, and curbing the loss of biodiversity”, the probability of forest land converting into cultivated, orchard, water, grassland, and construction land in the future will be reduced by 50%. The probability of grassland, orchard and water turning into forest land will increase by 30%. The probability of cultivated, orchard, grassland, and water area converting into construction land will decrease by 20%, and rivers and lakes are designated as ecological restricted areas.

#### 4.3.3. Landscape Ecological Risk Simulation

Compared with 2018, the area of CMA cultivated land, forest land, orchard and grassland will decrease in 2026 under the NDS (Table 5); the area of forest land will decrease by 23.80 km^2^, the area of construction land and water area will increase, and the area of construction land will increase by 27.91 km^2^, which is 84.96% of that in 2000–2018. The overall ERI value was 0.10036 in the simulation, significantly higher than the 2018 ERI. Compared with 2018, the area of higher and the highest risk increase by 397.5 km^2^, mainly from the lowest-risk areas (Table 6). This is because the trend of rapid urbanization continues under the NDS. In the simulation, the area of urban construction land and aquaculture ponds increase, and cultivated, orchard, forest, and grassland become occupied. In particular, the reduction in forest land disperses the landscape patches, destroys the integrity of the landscape, and leads to an increase in the ecological risk of the landscape.

**Figure 6:**
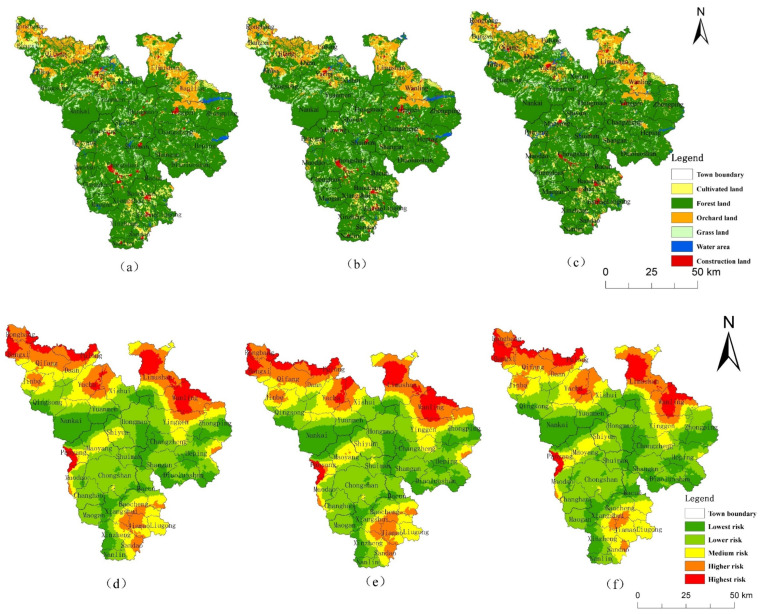
Multi-scenario simulation of land-use (**a**) NDS, (**b**) EDS, and (**c**) EPS, and landscape ecological risk (**d**) NDS, (**e**) EDS, and (**f**) EPS in 2026.

Compared with 2018, the main feature of land-use change under EDS in 2026 is a more dramatic expansion of urban construction land (Figure 6b). This is because SDG 9 and SDG 12 emphasize improving disaster prevention and mitigation infrastructure, residential safety, and industrial development. Correspondingly, the overall ecological risk also increased significantly in the simulation, with an ERI value of 0.101 (Figure 6e).

**Figure 7:**
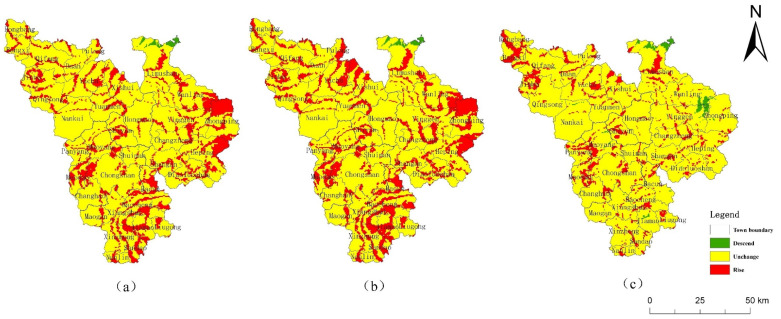
Spatial changes of landscape ecological risk from 2018 to 2026. (**a**) NDS, (**b**) EDS, (**c**) EPS.

Under the EPS, the expansion of construction land in 2026 is suppressed (Figure 6c), with an increase of only 17.04 km^2^. The water area increases by 1.07 km^2^, and aquaculture will be effectively controlled. The ecological land, especially the forest land, will be protected, with the area increasing by 31.6 km^2^. The ecological risk is reduced, and the ERI value was 0.09581. The ERI was higher than in 2018, but significantly lower than the first two scenarios (Figure 7), which is in line with the development goals of SDG 15 as a whole.

**Figure 8:**
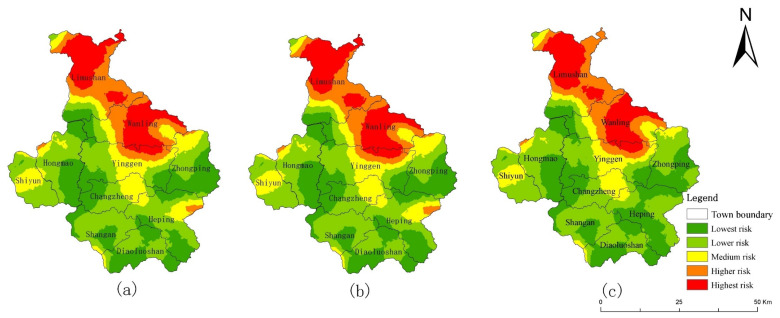
Multi-scenario simulation of landscape ecological risk (**a**) NDS, (**b**) EDS, and (**c**) EPS in 2026 of Qiongzhong County.

This study also simulated the future ecological risk of Qiongzhong County in 2026 with the same target scenario setting of the SDGs (Figure 8). Compared with 2018, the future ecological risks of the three scenarios in Qiongzhong increase. The ERI of NDS, EDS and EPS are 0.09495, 0.09555 and 0.094, respectively, and the relevant conclusions are consistent with the CMA. In the NDS and EDS simulation, the reduction in forest land accounts for more than 50% of the reduction in forest land in the CMA. Therefore, Qiongzhong County is a region with a high intensity of human activities in the CMA, and its ecological risk management is the key point to realize the SDGs of the CMA.

## 5. Discussion

This study combined SDGs to simulate regional land-use changes and explored the future ecological risk changes and the feasible path of SDGs in the CMA.

The research shows that the landscape ecological risk in most regions of the CMA is low, and the regional ecological background is good. High ecological risk areas are mainly distributed in low-altitude areas such as the northern, southeastern and central valleys of the CMA, mainly including orchard, cultivated, and construction land, which are areas with high intensity of human activities. From 2000 to 2018, urban expansion and economic forest planting encroached on natural forest land, leading to landscape fragmentation, that is, the negative disturbance of human activities in the study area led to an increase in ecological risks. However, under the influence of the continuous conversion of farmland to forests, the increase trend in ecological risks was slowed down; these conclusions are consistent with other research results [16]. Due to the strong connectivity of natural forest land and the enormous area of landscape patches, the fragmentation of the landscape is low. The natural forest land in the CMA is an important guarantee for maintaining regional ecological security. Therefore, in future planning, the intensity and scale of construction and development should be controlled in the high ecological risk areas of the CMA to avoid the further spread of high ecological risk areas. Strict forest protection policies should be planned to ensure the integrity and stability of the forest ecosystem and reduce ecological risks.

At present, many studies have explored the relationship between SDGs and water resources [29], land [30], energy, and food [31], but there are few studies on SDGs combined with landscape ecological risk. This study uses scenarios to predict the comprehensive evolution simulation of SDGs and landscape ecological risk from a spatial perspective. In the multi-scenario simulation, both the NDSs considering no SDG objectives and the EDSs considering the SDG 12 and SDG 9 objectives show that, in 2026, the expansion of cities and towns in the CMA will intensify and the ecological land will decrease, which will lead to increased ecological risk, and the ecological protection objectives of SDG 15 will be difficult to achieve, which is not conducive to the sustainable development of the region. Compared with the previous two scenarios, the ecological protection scenario considering the SDG 15 targets showed that the expansion of construction land has been suppressed, ecological land has been effectively protected, and the area of forest land has rebounded significantly. In addition, as a national key ecological function zone, to prevent the degradation of ecological functions, the CMA strictly controls development intensity, which also restricts local economic development and further increases the regional development gap with coastal cities and counties. Although the ecological protection scenario satisfies the goals of SDG 15, other goals including recent work and economic growth in SDG 8, the reduction in inequality in SDG 10, and inclusive and orderly urban construction outlined in SDG 11 are still difficult to achieve. The comprehensive scenario simulation results can aid a better understanding of the tradeoff and synergy among the sustainable development goals in the CMA. Studies have shown that the full use of synergies among SDGs, or the adoption of inclusive development, can bring economic, social, and environmental benefits [32]. Therefore, based on the positioning of key ecological function areas of the CMA, we should improve and implement the ecological compensation system, reduce the interference of human activities to the ecological space, and ensure the goal of ecological protection. Secondly, it is suggested that the CMA should develop eco-friendly industries, tap the potential of eco-tourism and healthcare, and enhance regional economics. Thirdly, priority should be given to the construction of a rural infrastructure system, improving the supply of basic public services in rural areas, and more importantly, raising public awareness of ecological civilizations [33]. These measures are conducive to improve the well-being of residents and better realize the SDGs.

This study used the construction of a landscape pattern index to evaluate the ecological risk of the CMA, which is of great significance to land-use optimization and regional sustainable development, and is a feasible method. However, the impacts of climate, geomorphology, and policy on ecological risk assessment were not considered, so the results are not absolute. Because of the typical spatial heterogeneity and scale effect of the landscape pattern index, some studies also show that the calculation of landscape index will deviate due to different spatial scales [34,35]. The scales selected in this paper were all within the range of 2–5 times the average area of the patches in the study area. The study shows that the scales within this interval had no significant impact on the results, and the ecological risks of each year were consistent. The difference is that a smaller grid scale can highlight the risk differences between different years. This study did not select the administrative scale because it is larger than the range, but the scale effect of landscape ecological risk needs to be further explored. Due to space limitations, not all the scale research details are listed. The final result shows that the 3 km × 3 km grid unit scale in this paper is suitable for the CMA’s landscape ecological risk assessment.

## 6. Conclusions

The land-use data of 2000, 2010, and 2018 were used to assess the landscape ecological risk in the CMA. The multi-scenario landscape ecological risk changes were simulated combined with the SDGs, which provided a basis for implementing SDGs and regional sustainable development. The relevant conclusions are as follows.

The temporal and spatial distribution of the ecological risk in the CMA was quantitatively analyzed through the landscape ecological index. The lower ecological risk area and the low ecological risk area were mainly distributed in the central part of the CMA. Forest land was found to be the primary land use in the CMA, accounting for over 74% of the total. The forests have strong connectivity and large patches, which provide important support for the ecological security of the CMA. The higher and highest ecological risk areas were at the edge of the CMA, a region with an obvious intensity of human activities. The area of construction land in the CMA nearly doubled from 2010 to 2018, and occupied forest land, which was the main reason for the increase in ERI in the CMA. However, due to the continuous policy of returning farmland to forest, the ERI of CMA can be effectively limited. In the ecologically high-risk areas of Qiongzhong and Baisha County, it is necessary to limit the scope and intensity of human activities to prevent the further spread of ecological risks from the edge to the center in CMA. Therefore, in order to ensure ecological security, it is necessary to: control the expansion of construction land; reduce the encroachment on ecological land by human activities; to protect the regional ecological environment; to improve the ability of the environment to resist ecological environment risks; and to maintain ecological security.

The PLUS model has a good coupling mechanism with land-use changes triggered by social, economic, and environmental factors, and the model has a high simulation accuracy, which gives it good explanatory properties in different scenario simulations. Therefore, it can be used as an effective research tool for SDG scenario simulation to provide policy-making suggestions. By combining the analysis of the interaction between SDGs and landscape ecological risks, a landscape ecological risk monitoring simulation based on localized SDGs was constructed, and a land-use planning system was implemented based on the evaluation results in order to provide a practical basis for the application of SDGs in terms of regional economic, social, and ecological civilization construction.

By combining the analysis of the interaction between SDGs and landscape ecological risks, a landscape ecological risk monitoring simulation based on localized SDGs was constructed, and a land-use planning system was implemented based on the evaluation results in order to provide a practical basis for the application of SDGs in terms of regional economic, social, and ecological civilization construction.

## Figures and Tables

**Table 1:** Sustainable development goals.

Goals	Subject	Specific Objectives
SDG 1	No Poverty	End poverty in all its forms, everywhere
SDG 2	Zero Hunger	End hunger, achieve food security and improved nutrition, and promote sustainable agriculture
SDG 3	Good Health and Well-Being	Ensure healthy lives and promote well-being for all at all ages
SDG 4	Quality Education	Ensure inclusive and equitable quality education and promote lifelong learning opportunities for all
SDG 5	Gender Equality	Achieve gender equality and empower all women and girls
SDG 6	Clean Water and Sanitation	Ensure availability and sustainable management of water and sanitation for all
SDG 7	Affordable and Clean Energy	Ensure access to affordable, reliable, sustainable, and modern energy for all
SDG 8	Decent Work and Economic Growth	Promote sustained, inclusive and sustainable economic growth, full and productive employment, and decent work for all
SDG 9	Industry, Innovation, and Infrastructure	Build resilient infrastructure, promote inclusive and sustainable industrialization, and foster innovation
SDG 10	Reduced Inequalities	Reduce inequality within and among countries
SDG 11	Sustainable Cities and Communities	Make cities and human settlements inclusive, safe, resilient, and sustainable
SDG 12	Responsible Consumption and Production	Ensure sustainable consumption and production patterns
SDG 13	Climate Action	Take urgent action to combat climate change and its impacts
SDG 14	Life Below Water	Conserve and sustainably use the oceans, seas, and marine resources for sustainable development
SDG 15	Life on Land	Protect, restore, and promote sustainable use of terrestrial ecosystems; sustainably manage forests; combat desertification; halt and reverse land degradation; and halt biodiversity loss
SDG 16	Peace, Justice, and Strong Institutions	Promote peaceful and inclusive societies for sustainable development; provide access to justice for all; and build effective, accountable, and inclusive institutions at all levels
SDG 17	Partnerships for the Goals	Strengthen the means of implementation and revitalize the global partnership for sustainable development

**Table 2:** Calculations of the landscape index and ecological meaning.

Index	Computation	Ecological Meaning of Index
Landscape fragmentation (*Ci*)	$C_{i}=\frac{n_{i}}{A_{i}}$	It indicates the process of land-use type changing from continuous whole patch to complex discontinuous patch under natural or human disturbance. The larger the value is, the lower the stability of the corresponding land-use ecosystem is. *n*_i_ is the number of patches of land-use type *i*.
Landscape separation (*Ni*)	$N_{i}=\frac{1}{2}\sqrt{\frac{n_{i}}{A}}\times \frac{A}{A_{i}}$	It indicates the degree of separation between different patches in the landscape type. The larger the value is, the more complex the spatial distribution of the land-use type is and the higher the separation degree is. *A_i_* is the area of land-use type *i* and *A* is the total area of landscape.
Landscape fractal dimension (*Fi*)	$F_{i}=\frac{2\ln\left(P_{i}/4\right)}{\ln A_{i}}$	The value range of *F_i_* is 1–2. The larger the value, the more complex the shape of land-use patches. When *F_i_* < 1.5, the patch shape is relatively simple; when *F_i_* =1.5, the patch is in Brownian random motion state, with poor stability; when *F_i_* > 1.5, the patch shape is complex. *P_i_* is the perimeter of land-use type *i*.
landscape vulnerability index (*V_i_*)	Obtained by normalization	Based on the relevant research [24,25] and combined with the landscape pattern characteristics in the CMA, the landscape vulnerability was divided into five levels from low to high: 5—water; 4—cultivated and orchard; 3—grassland; 2—forest land; 1—construction land.

**Table 3:** Land-use changes in CMA from 2000 to 2018.

Year	Cultivated Land (km^2^)	Forest Land (km^2^)	Orchard (km^2^)	Grassland (km^2^)	Water Area (km^2^)	Construction Land (km^2^)
2000	599.16	5264.29	733.58	440.33	49.47	28.57
2010	592.63	5308.71	716.57	408.68	57.41	31.42
2018	583.70	5286.44	707.12	407.00	69.74	

**Table 4:** The results of landscape pattern index.

Land Use	Year	Number of Patches	Area	Fragmentation Index (C_i_)	Separation Index (N_i_)	Fractal Dimension Index (F_i_)	Disturbance Index (E_i_)
Cultivated land	2000	1398	59,916	0.0233	0.2632	1.1040	0.3114
	2010	1311	59,263	0.0221	0.2577	1.1104	0.3104
	2018	1318	58,370	0.0226	0.2623	1.1101	0.3120
Forest land	2000	400	526,429	0.0008	0.0160	1.0773	0.2206
	2010	380	530,871	0.0007	0.0155	1.0815	0.2213
	2018	419	528,644	0.0008	0.0163	1.0828	0.2219
Orchard	2000	481	73,358	0.0066	0.1261	1.0901	0.2591
	2010	490	71,657	0.0068	0.1303	1.0884	0.2602
	2018	493	70,712	0.0070	0.1324	1.0906	0.2613
Grassland	2000	1242	44,033	0.0282	0.3376	1.1031	0.3360
	2010	1216	40,868	0.0298	0.3599	1.1043	0.3437
	2018	1212	40,700	0.0298	0.3608	1.1036	0.3438
Water area	2000	172	4947	0.0348	1.1181	1.1102	0.5748
	2010	162	5741	0.0282	0.9351	1.1125	0.5172
	2018	175	6974	0.0251	0.8000	1.1135	0.4752
Construction land	2000	224	2857	0.0784	2.2092	1.0561	0.9132
	2010	225	3142	0.0716	2.0133	1.0586	0.8515
	2018	314	6142	0.0511	1.2168	1.0596	0.6025

**Table 5:** Land-use change under different scenarios.

	Cultivated Land (km^2^)	Forest Land (km^2^)	Orchard (km^2^)	Grassland (km^2^)	Water Area (km^2^)	Construction Land (km^2^)
2018	583.70	5286.44	707.12	407.00	69.74	61.42
2026 NDS	575.63	5262.72	698.11	405.11	80.78	89.32
2026 EDS	572.12	5256.61	695.59	404.67	80.71	101.97
2026 EPS	557.52	5318.12	690.24	392.89	74.45	78.44
2018–2026 NDS	−8.06	−23.80	−9.02	−1.90	11.12	27.91
2018–2026 EDS	−11.58	−29.91	−11.54	−2.35	11.04	40.57
2018–2026 EPS	−22.45	31.6	−16.88	−14.12	1.07	17.04

**Table 6:** Area changes of different levels of ecological risk in the CMA.

Year	Lowest Risk (km^2^)	Lower Risk (km^2^)	Medium Risk (km^2^)	Higher Risk (km^2^)	Highest Risk (km^2^)
2000	1667.46	2623.59	1447.02	1020.31	356.33
2010	1860.46	2472.42	1391.25	1018.54	372.05
2018	1902.95	2429.04	1381.07	1006.37	395.29
2026 NDS	1405.64	2528.86	1448.13	1183.43	548.66
2026 EDS	1370.89	2512.48	1416.04	1207.77	607.54
2026 EPS	1647.77	2537.71	1408.07	975.60	545.57
2018–2026 NDS	−497.32	99.82	67.06	177.06	153.38
2018–2026 EDS	−532.07	83.44	34.97	201.41	212.25
2018–2026 EPS	−255.19	108.67	27.00	−30.76	150.28

## Data Availability

The data presented in this study are openly available in Resource and Environmental Science Center of the Chinese Academy of Sciences (www.resdc.cn, 1 December 2020), National Basic Geographic Information Center (www.ngcc.cn/ngcc, 1 December 2020) and Hainan Statistical Yearbook.
